# A Retrospective Investigation of Abortion Storm in Abergele Goats, Waghimira Zone, Amhara Region, Ethiopia

**DOI:** 10.1155/2024/5686443

**Published:** 2024-09-05

**Authors:** Adane Bahiru, Ayalew Assefa, Biruk Alemu Gemeda, Hiwot Desta, Abebe Tibebu, Abebe Sahle, Barbara Wieland

**Affiliations:** ^1^ Sekota Dry-Land Agricultural Research Center, P.O. Box 62, Sekota, Ethiopia; ^2^ International Livestock Research Institute, P.O. Box 5689, Addis Ababa, Ethiopia; ^3^ Ziquala Woreda Office of Agriculture, Ziquala, Ethiopia; ^4^ Institute of Virology and Immunology, Mittelhäusern, Switzerland; ^5^ Department of Infectious Diseases and Pathobiology Vetsuisse Faculty University of Bern, Bern, Switzerland

## Abstract

A retrospective study was conducted in Abergele and Ziquala districts in Ethiopia to investigate the occurrence, impact, and potential risk factors for abortion in small ruminants linked to a regional abortion storm. Affected (case) and nonaffected (control) villages were compared to assess infectious and noninfectious risk factors causing abortions. A case village was defined as a village with abortion seen in all households enrolled in the study, while a control village is characterized by presence of abortion in two and fewer households. A questionnaire survey, focus group discussions, and serological examinations were used to assess the differences in the abortion rate between the case and control villages. The Rose Bengal Plate Test for Brucellosis, *Toxoplasma gondii* Antibody Test Kit for *Toxoplasma gondii*, and ELISA for *Chlamydophila abortus* and *Coxiella burnetii* were used to detect antibodies. Per village 15 household flocks were selected. In the case villages, all flocks were affected by abortion (a mean abortion of 13 animals in Abergele and 9 in Ziquala). In contrast, only three households reported abortions in the control villages. A total of 176 blood samples were collected from the case and control villages for further laboratory diagnosis of possible causes of abortion. Of the examined flocks, 17%, 10.2%, and 2.8% were positive for *Coxiella burnetii*, *Toxoplasma gondii*, and *Chlamydophila abortus,* respectively. However, antibodies against *Brucella melitensis* were not detected. While the seroprevalence was greater for some infectious agents of abortion, there was no significant difference between the case and control villages. It is clear that the abortion problem in the study villages is complex and likely due to a mix of poor husbandry practices and the presence of infectious diseases. To better understand the underlying causes of abortion, there is a need to conduct a longitudinal study involving testing for more pathogens at the household level combined with reliable data on husbandry practices.

## 1. Introduction

Sheep and goats are highly adaptable to a broad range of environmental conditions [1]. Moreover, their low cost of production, low land requirements, and high prolificacy make them attractive assets for smallholder households [2]. Investment in sheep and goats reduces losses due to the high inflation rates that are found in unstable economies of many developing countries such as Ethiopia [3]. Ethiopia is an agriculturally based country with a considerable number of small ruminants, estimated to be more than 61 million heads [4]. The Abergele goat is a known goat breed in Ethiopia with an estimated population of more than 300,000 and making up to approximately 75% of the liquid cash income for smallholder farmers in the community [5]. Despite the large small ruminant population sizes, the country fails to optimally utilize these resources, as the sector suffers reduced productivity due to various factors in which diseases stand on the front line. One of the key animal health challenges for productivity of small ruminants is reproductive failure, which has major economic, animal welfare, and public health impacts [3].

Broadly, causes of reproductive failure are categorized into infectious and noninfectious causes [6]. The effects on sheep and goat breeding are abortion, stillbirth, and delivery of weak lambs or kids [7, 8]. This leads to less replacement animals, reduced milk production, and a large number of animals with extended unproductive seasons [9]. A retrospective study in Ethiopia showed the presence of higher losses associated with abortion in 29.8% of goats under study [10].

Additionally, abortion in sheep and goats has become increasingly important because of the potential zoonotic significance of commonly involved pathogens, such as *Coxiella burnetii* and *Chlamydophila abortus* [11]. Toxoplasmosis is also a potential causative agent of abortion and is a zoonosis [12]. Another important infectious disease that causes abortion in sheep and goat is *Brucella melitensis* [13]. A study in Ethiopia revealed that *Coxiella burnetii*, *Chlamydophila abortus*, *Toxoplasma gondii*, and *Brucella* species known to be the cause of abortion in small ruminants are detected serologically [14].

*Coxiella burnetti*, the causative agent of Q-fever, is a bacterial disease that can infect humans as well as a wide range of animals. The disease is characterized by fever and can cause abortion in dairy animals [15].

*Chlamydiae* are obligate intracellular bacteria, which have higher prevalence in animals. They infect ocular, genital, and respiratory tissues leading to a variety of diseases including abortion, pneumonia, gastroenteritis, encephalomyelitis, kerato conjunctivitis, arthritis, orchitis, seminal vesiculitis, and epididymitis [16].

Brucellosis is an infectious disease of worldwide importance in domestic ruminants, and the causative bacteria (*Brucella abortus* and *Brucella melitensis* in sheep and goats) are transmitted to humans through contact with infected or by consumption of contaminated dairy products [13].

Toxoplasmosis is a parasitic disease of humans, wild and domestic animals caused by *Toxoplasma gondii*, a cosmopolitan protozoan of the sarcocystidae family with animals of the Felidae family as a definitive host. Toxoplasmosis is characterized by signs of hyperthermia, dyspnea, and neurological problems. It is considered as a major causative agent of abortion is also a zoonosis disease [12]. The difference in climatic and geographical factors and closeness of human and animal contacts affects its prevalence [9]. The disease can transmit between animals through the ingestion of contaminated oocytes. Besides, it can also be transmitted congenitally [17]. Consumption of tissue cysts from undercooked meat and oocysts' intake from food is a possible source of infection for humans.

Abortion is probably the most common animal health challenge in goats in the Abergele and Ziquala districts of the Amhara region in Ethiopia. The consequence of an abortion storm can have a devastating economic impact on the community where goat production is the basis for livelihood. Given the recently reported increase in abortion incidence with significant differences in the magnitude of abortion across villages, a detailed investigation was conducted to assess the occurrence and magnitude of abortion in case and control villages and to identify risk factors and potential pathogens responsible for abortion through serological methods.

## 2. Materials and Methods

### 2.1. Description of the Study Area

**Figure 1:**
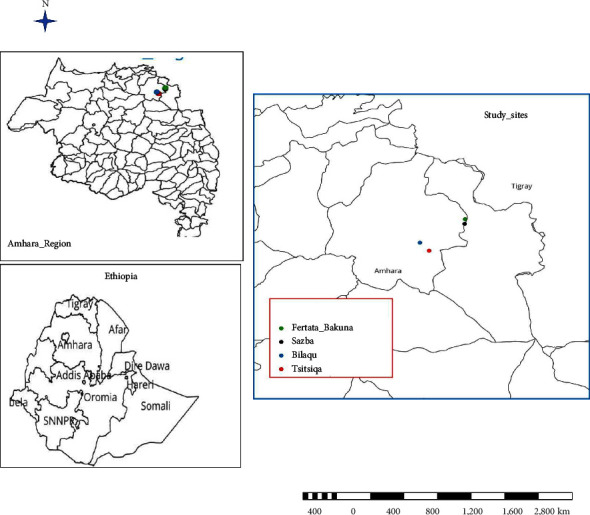
Location map of the study sites.

The study was carried out in two selected districts (Ziquala and Abergele) of the Amhara region, Northern Ethiopia (Figure 1). The study sites were selected purposely based on the reports of abortion outbreaks. The districts are warm submoist lowlands and are located at an average altitude of 1450 masl. The average annual rainfall varies from 350 to 650 mm, and the temperature ranges from 18 to 42°C [18].

### 2.2. Study Design, Sampling, and Definition of Cases and Controls

A retrospective study was conducted to determine differences in the magnitude of abortion between the case and control villages and to identify the prevalent pathogens. Cases were defined as villages with recent large abortion storms (Sazba and Bilaqu) where all households in the village are encountered with abortion, whereas controls were adjacent villages (Fertata bakuna and Tsitsiqa) with relatively where few (less than two) households were with the incident. A total of 176 blood samples were collected for further laboratory diagnosis. Three animals per household one male serving as a mating buck and two female does who gave birth at least once were included in the sampling.

### 2.3. Questionnaire Survey and Focus Group Discussions (FGD)

A questionnaire survey was administered to a total of 58 households, and 4 FGDs were conducted with small ruminant producing farmers. About 30 of them were drawn from two case villages (Sazeba in Abergele district and Bilaque in Ziquala district), and 28 of them were drained from two control villages (Tsitsika in Ziquala district and Fertata Bakuna in Abergele district).

### 2.4. Sample Collection

Blood samples were collected by bleeding from the jugular vein using vacutainer needles with their respective plastic cap and adjusted to 8 ml vacuum tubes for serum. The tubes were transported in coolers at temperatures between 5 and 10°C. Blood samples were allowed to stand for 24 hours at room temperature for serum separation. The serum was frozen at −20°C until laboratory procedures were performed.

### 2.5. Laboratory Procedures

#### 2.5.1. Rose Bengal Plate Test

A Rose Bengal plate test kit at National Animal Health Diagnostic and Investigation Center (NAHDIC), Sebeta, Ethiopia, was used to detect *Brucella melitensis* bacteria-specific antibodies in the goat serum samples. The antigen, test, and control sera were allowed to cool at room temperature for 30 minutes before use, followed by gently shaking the antigen bottle to obtain a homogeneous suspension. Finally, 25–30 *μ*l of serum and antigen were placed on a plate, and the serum and antigen were mixed thoroughly and rapidly. The mixture was shaken for approximately four minutes, and the formation of agglutination was observed. The results were expressed as no agglutination (negative), visible agglutination (positive), and flocculates (false agglutination) as interpretable or unreadable [19].

#### 2.5.2. Enzyme-Linked Immunosorbent Assay (ELISA)

Direct ELISA from IDEXXR Switzerland AG, 3097 Liebefeld Bern Switzerland, was used to detect specific antibodies for *Chlamydophila abortus* and *Coxiella burnetii*. Serum samples were processed according to the manufacturer's protocol. The sera were diluted 1 : 10 for the analysis of each agent. For adequate interpretation, the average optical density (OD) of the positive controls and the difference between the ODs of the positive and negative control sera were calculated. Serum positive percentages (S/P) were calculated according to OD data from the different serum samples and the average OD of the positive control sera using the following formula: S/P = (OD of sample × 100): (average OD of positive control). As recommended by the manufacturer, serum samples with S/P percentages of 50% were considered positive [20].

For Toxoplasma, the indirect ELISA test from IDEXXR Switzerland AG, 3097 Liebefeld-Bern Switzerland, was used to detect antibodies against *Toxoplasma Gondi*. The antigen is coated onto the solid phase, and the sample containing antibodies is added. The antigen-antibody reaction is enhanced by the addition of a secondary enzyme-linked antibody, and the reaction can be evaluated by quantification of the color that develops [21].

### 2.6. Data Management and Analysis

All the records were coded in Microsoft Excel, and analysis was performed using the STATA 14 computer program. Descriptive and inferential statistics (chi-square test and logistic regression) were used to summarize the results. A *p* value less than 0.05 was considered to indicate a statistically significant association between variables.

## 3. Results and Discussion

### 3.1. Background Characteristics of the Participants

Most of the respondents (86.2%) were males, and 72.4% of them could not read or write (Table 1). This result is in line with previous reports conducted in the same study area where more males than females participated in household surveys [22], and a significant number of them were unable to read and write [18]. This is not surprising, as most household activities are under the command of males, and educational coverage is considerably lower than that in other parts of the country.

### 3.2. Small Ruminant Herd Structure of Surveyed Households

The herd structure indicates that the proportion of young (particularly the yearlings) stock was lower than the number of adults (Table 2). This confirms the finding of other studies in Ethiopia, where reproductive ages constituted the largest share of the goat population [23, 24]. This is associated with the need to have many offspring by maintaining reproductive ewe and doe compared with males and other age groups. The other reason is associated with off-take, where yearlings are mostly brought to market, consumed at home for festivities and shared with herders, making them have a relatively lower share of the herd structure.

### 3.3. The Occurrence of Abortion and Differences Noted by Farmers in Case and Control Villages

Abortion problems were normally reported every year; however, farmers described an increased number of abortions in 2016. The abortion storm was reported explicitly in goats, except for very few abortions in sheep and cattle, which were not significant in number. All 30 household flocks included in the case villages were affected by abortion with different magnitudes compared to those of the control villages, which reported only three households out of 28. It was evident that the scope of abortion is much greater in case villages since the cases and controls are purposely defined. A total of 339 animals from the study sites, 327 from the case sites, and 12 from the control villages aborted. On average, 13 sheep/goats aborted per household in Sazeba village, with a minimum of 3 and a maximum of 30 ewes/doe per household (Table 3). This might be associated with differences in the number of pregnant does and ewes. During the FGD, farmers in the control villages indicated that they had a water source in the form of a river that was found near their village, and the provision of supplementary feed was commonly practiced. Furthermore, the households in the case villages had on average larger herds.

### 3.4. Months of the Year with Higher Episodes of Birth and Abortion

**Figure 2:**
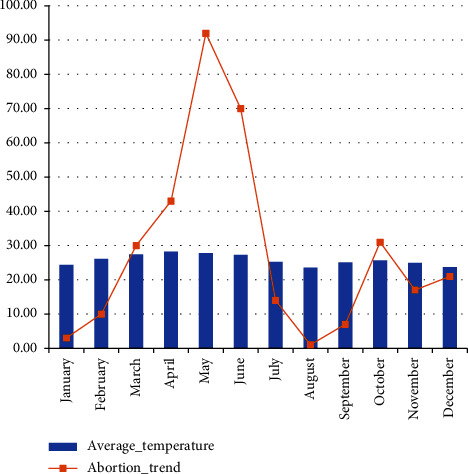
Distribution of abortion and average temperature across months of the year in 2016.

**Figure 3:**
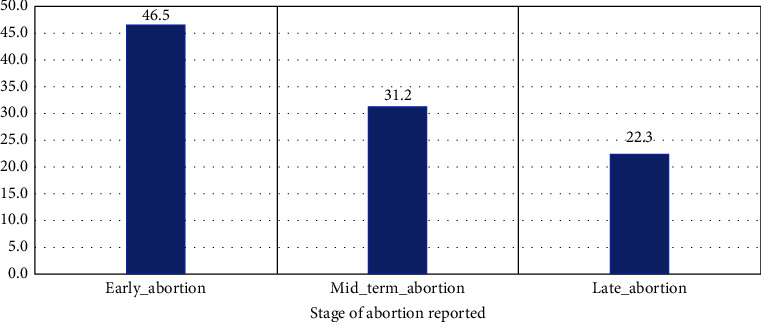
Distribution of abortion across stages of pregnancy.

During the FGD, farmers reported that abortion storms occurred in two periods (Figure 2). The first phase of abortion occurred between March and June, with a peak in May, 2016. Many abortions were challenging to detect since they only saw blood and noted vaginal discharge, indicating early abortions. The March-June season is known in the area as the long dry season, with high temperatures and degraded feed resources. These factors are the most common causes of abortion [14]. According to the respondents, 46.5% of abortions occurred during the early phase of pregnancy, less than one month, while 22.3% reported abortion during the late stage of pregnancy (Figure 3). In the study area, a minor rainfall episode from late January to February led to green vegetation making sheep and goats to be in relatively better environments and subsequently become pregnant. However, subsequent dramatic changes to harsh environmental situations make them unable to support pregnancy, and early abortion can occur.

The second phase of abortion occurred from October to November 2016. They described this phenomenon as late abortion since kidding/lambing is mostly expected in November and December. They reported large fetuses; some were full-term kids with skin cover. The season from October to December is known as the season of the year where the largest shares of kids are born [24]. The abortion storm from October to December was significantly lower than its occurrence from March to June. This might be associated with the availability of feed resources for grazing and remnants in crop farmlands and sufficient watering sources at a reasonable distance.

### 3.5. Causes and Clinical Signs of Abortion and Management of Aborted Fetuses by Farmers

The commonly mentioned clinical signs in aborting animals were rough hair coats, fever, vaginal discharge, and retained placenta. About 29.73% and 37.84% of the respondents reporting abortion also experienced stillbirths and weak kids/lambs, respectively, during the abortion outbreak. There was no practice of proper disposal of the fetus after an abortion. The majority of respondents, 80.95%, stated that they had fed aborted fetuses to their dogs, 33.33% of them left aborted fetuses in the surroundings, and only 2.38% of the respondents reported burying aborted material (Table 4). This result is comparable to that of a study in Yabello, Ethiopia, where the largest portion of respondents did not have knowledge about the dissemination of disease associated with improper disposal of aborted fetuses [25]. Further, there are no health intervention programs available targeting the reduction of small ruminant abortion. These conditions might be the reason for the continued occurrence of abortion in the study areas.

During FGD, farmers suggested that the cause of the abortions in October-December was disease (Table 4). They mentioned the occurrence of Peste Des Petits Ruminants (PPR) and sheep and goat pox in some flocks based on clinical signs. They noted that weather conditions, feed, and water availability were relatively pleasant during this time due to the main rainy season. In contrast, most farmers relate abortions from March to June to the drought season (harsh sun), which affected the animals in the weeks after mating. They mentioned “*Tegage*,” a weather condition characterized as very hot and dull air. This weather occurs approximately every 2 or 3 years and causes drying of the vegetation, shrubs, and trees. There was no change in the water source or animal movement compared to previous years. Approximately 53% of the respondents thought that the cause of abortion was the extended dry season, drought and feed scarcity, and environmental stress due to intense sunlight. Only 12.5% assumed that the cause could be disease. This result is in line with a previous study on sheep and goat abortion, where 56% of participants described extreme weather conditions and feed shortages as causes of abortion [14].

There were some husbandry management practices in place to minimize abortion. Approximately 59.4% of respondents described that during times of abortion storms, they try to keep pregnant animals around the homestead in the shed to protect them from direct sunlight and supplement them with crop residues such as pearl millet and sorghum stover, and 56.25% of them believe this helped greatly reduce the abortion level. Although a considerable proportion of the participants believe that additional feed sources are important in limiting abortion, they still did not practice due to a lack of feed available for managing large flocks.

### 3.6. Infectious Causes of Abortion

Serological investigations of samples collected from aborted animals indicated that *Coxiella burnetii* (17%), *Toxoplasma gondii* (10.2%), and *Chlamydophila abortus* (2.8%) were possible causes. The overall prevalence of organisms in both the case and control groups is depicted in the following table (Table 5).

Logistic regression analysis of the occurrence of organisms in both the case and control groups revealed that there was no significant difference between the sex groups and study districts. However, the occurrence of *Coxiella burnetii* differed substantially between the two study districts (*p*=0.017). Its occurrence was much greater in Abergele than in Ziquala district (Table 6).

*Brucella melitensis,* a well-known cause of small ruminant abortion, was not detected in this particular study area in neither the case or control group. The findings of this study were in line with those of a study conducted in Menze and Horro small ruminants. In these locations, the prevalence of small ruminant brucellosis was also zero [26]. In contrast, Brucellosis in small ruminants has been reported in Yabello district in pastoralist systems [27] where the overall incidence was 1.88% and a meta-analysis indicated that the overall pooled prevalence of brucellosis in goats in Ethiopia was 2.7% [28]. This finding is not surprising, as the incidence/prevalence of brucellosis is decreasing in Ethiopia and is almost insignificant in many studies. Generally, the study revealed the presence of infectious causes of abortion; however, there was no significant difference in their occurrence between the case and control groups. Therefore, the main reason for the differences in the magnitude of abortion might be associated with differences in husbandry practices in response to harsh environmental conditions.

## 4. Conclusions

The study showed that abortion in the enrolled villages is complex and likely involves a mix of poor husbandry practices and the presence of infectious diseases. However, the study did not investigate all possible abortion pathogens. Farmers in the FGD greatly accepted the importance of supplementary feeding, as a fraction of them were practicing for improvement of management practices. Improving feeding and goat management practices to reduce abortions related to feed shortages and climate stress is highly recommended and clearly would help increase the resilience of small ruminants. To better understand the underlying causes and relative importance of the husbandry system, we recommend conducting a longitudinal study that confirms the cause of abortion with community participation in a timely manner combined with household-level data collection. Additionally, measures need to be taken to enhance the practice of supplementary feed provision during harsh environmental conditions, vaccination for prevailing diseases that induce abortion, proper disposal of aborted materials, and isolation and culling of repeatedly involved animals.

## Figures and Tables

**Table 1:** Demographic characteristics of the participants.

	Demography of participants	*N* (%)
Sex	Female	8 (13.79%)
	Male	50 (86.20%)

Age of respondent	≤30	13 (22.41%)
	31–45	25 (43.10%)
	≥46	20 (34.48%)

Education level	Can't read and write	42 (72.41%)
	Can read and write	10 (17.24%)
	Formal education	6 (10.34%)

**Table 2:** Herd structure owned by study participants in Ziquala and Abergele districts.

District	Kebele	Status	Mean number of					Mean number of				
			Total sheep	Ewes	Lambs	Yearlings	Rams	Total goat	Does	Kids	Yearlings	Bucks
Abergele	Sazeba	Case	15	8	9	2	2	50	32	16	3	2
	Fertata baquna	Control	16	9	3	2	2	28	15	7	5	1

Zequala	Bilaque	Case	18	8	6	3	1	33	18	10	5	2
	Tsetseka	Control	8	3	2	1	1	11	6	3	1	1

**Table 3:** Number of abortions reported in Abergele and Ziquala districts.

District	Kebele	Number of households with aborted goat and sheep	Status	Mean no. of animals aborted per household	Min	Max	Total no. of animals aborted
Abergele	Sazeba	15	Case	13	3	30	193
	Fertata baquna	1	Control	0.3	0	5	5

Ziquala	Bilaque	15	Case	9	1	20	134
	Tsitsika	2	Control	0.5	0	5	7

							339

**Table 4:** Causes of abortion, signs and management of aborted fetuses.

Variables	Responses	*N* (%)
What do you think was the reason for abortions encountered	Feed scarcity during long dry season	09 (28.13%)
	Climatic stress	09 (28.13%)
	Don't know	10 (31.25%)
	Disease	04 (12.5%)

What do you do to prevent abortion	Do nothing	13 (40.63%)
	Protect from direct sunlight and feed supplement	19 (59.38%)

Are these preventive practices effective	No	14 (43.75%)
	Yes	18 (56.25%)

How do you dispose of aborted material	Burning	01 (2.38%)
	Burying	01 (2.38%)
	Feed to dogs	34 (80.95%)
	Nothing practiced/leave as it is	14 (33.33%)

Abnormalities encountered during abortion	Still birth	11 (29.73%)
	Weak kids	14 (37.84%)
	Didn't recognize	12 (32.43%)

**Table 5:** Seroprevalence of isolated organisms in the Abergele and Ziquala districts.

Grouping variable	*Toxoplasma gondii* (*n* = 18/176)		*Coxiella burnetii* (*n* = 30/176)		*Chlamydophila abortus* (*n* = 5/176)	
	Cases	Controls	Cases	Controls	Cases	Controls
Sex						
Male	1	4	14	3	1	1
Female	6	7	5	8	1	2
District						
Ziquala	6	9	4	4	0	1
Abergele	1	2	15	7	2	2
Total (n/N)	7/88	11/88	19/88	2/88	3/88	6/88

**Table 6:** Association of seroprevalence with differences in sex and district.

	*Toxoplasma gondii*			*Coxiella burnetii*			*Chlamydophila abortus*		
	*p* value	OR	95% CI	*p* value	COR	95% CI	*p* value	COR	95% CI
Sex									
Female	Ref								
Male	0.55	0.72	0.24–2.12	0.34	0.65	0.27–1.58	0.77	1.29	0.21–7.99
District									
Ziquala	Ref								
Abergele	0.45	0.68	0.25–1.83	0.017^∗^	2.9	1.21–6.94	0.746	1.35	0.22–8.28
Status									
Cases	Ref								
Controls	0.228	1.61	0.74–3.48	0.384	0.72	0.34–1.52	0.843	1.15	0.28–4.77

OR, odds ratio; ^∗^indicates a significant difference at a *p* value of 0.05.

## Data Availability

The data will be made available upon request to the corresponding author.
